# Moral failure, moral prudence, and character challenges in residential care during the Covid-19 pandemic

**DOI:** 10.1177/09697330231174532

**Published:** 2023-06-09

**Authors:** Settimio Monteverde

**Affiliations:** School of Health Professions, 69477 Bern University of Applied Sciences, Bern, Switzerland; Institute of Biomedical Ethics and History of Medicine, University of Zurich, Zurich, Switzerland

**Keywords:** COVID-19, residential care, nursing homes, moral character, virtue ethics, moral distress

## Abstract

In many high-income countries, an initial response to the severe impact of Covid-19 on residential care was to shield residents from outside contacts. As the pandemic progressed, these measures have been increasingly questioned, given their detrimental impact on residents’ health and well-being and their dubious effectiveness. Many authorities have been hesitant in adapting visiting policies, often leaving nursing homes to act on their own safety and liability considerations. Against this backdrop, this article discusses the appropriateness of viewing the continuation of the practice of shielding as a moral failure. This is affirmed and specified in four dimensions: *preventability of foreseeable harm, moral agency, moral character,* and *moral practice* (in MacIntyre’s sense). *Moral character* is discussed in the context of prudent versus proportionate choices. As to *moral practice*, it will be shown that the continued practice of shielding no longer met the requirements of an (inherently moral) practice, as external goods such as security thinking and structural deficiencies prevented the pursuit of internal goods focusing on residents’ interests and welfare, which in many places has led to a loss of trust in these facilities. This specification of moral failure also allows a novel perspective on moral distress, which can be understood as the expression of the psychological impact of moral failure on moral agents. Conclusions are formulated about how pandemic events can be understood as character challenges for healthcare professionals within residential care, aimed at preserving the internal goods of residential care even under difficult circumstances, which is understood as a manifestation of moral resilience. Finally, the importance of moral and civic education of healthcare students is emphasized to facilitate students' early identification as trusted members of a profession and a caring society, in order to reduce experiences of moral failure or improve the way to deal with it effectively.

## Introduction

During the first wave of the Coronavirus pandemic in spring 2020, highly disturbing news, and images of the devastating impact of Covid-19 in residential care in many high-income countries received ample media coverage worldwide. As an immediate response, many health authorities and administrations of residential care facilities put into force more or less drastic restrictions or bans on visits.^1,2^ Given the scant epidemiological knowledge at that time, these measures were deemed necessary to best respond to the early emerging susceptibility of residents to severe and often deadly courses of Covid-19.^3,4^ However, as the pandemic continued, these measures have been increasingly questioned, given not only their harms in terms of health and quality of life for those affected, but also in terms of effectiveness.^5,6,7,8^

## The practice of shielding as an ethical first response to residents’ vulnerability

At the heart of justifying visiting bans stood the imperative to protect the lives of residents.^9^ The underlying justification combined different assumptions: First, that the gradually emerging vulnerability of residents was essentially *physical* and had therefore to be understood as an obligation to protect physical life. Second, that this protection should preferably be done by shielding the residents *inside* from all possible carriers of the virus “lurking” from the *outside*.^10^ And third, that restrictions on fundamental rights of residents, that is, persons living in their privately inhabited spaces within a healthcare facility,^11^ made these measures not only appear necessary and unavoidable, but also reasonable in the light of the perceived risk and imminent danger, even despite an initially scant evidence base.^8^ Besides this argument merging vulnerability with epidemiological and public health considerations, this first response—hereafter referred to as the *practice of shielding*—was also triggered psychologically: First, by the shock that the images spread in the media and social networks prompted showing somber processions of military trucks with coffins moving in the dark of night from residential care facilities to mortuaries. And second—given the scant knowledge about the virus at the beginning of the pandemic—by the authorities’ and facility management’s fear of being charged with breaches of due diligence.^12,13,14^

In retrospect, the first response to the pandemic resulted in a practice of shielding (whose most visible characteristic were bans on visits), based on the perception of situational risk to the residential care facility population and uncertainty about the dynamics of the virus. In parallel, excessive demands on health authorities and residential care facility managers also acted as trigger: not only those responsible were concerned with providing the best possible protection for a population characterized by frailty, dependency, and age-related morbidity, but also with avoiding legal consequences resulting from failing to create a sufficiently safe environment for the most vulnerable.^15^

However, in the successive phases of the pandemic, infection prevention measures have been constantly loosened in the public, with tests, vaccines, knowledge, and means of more effective protection being increasingly available. Many health authorities introduced only very hesitantly relaxations to visiting bans, for example, for residents at the end of life.^16,17,18^ Thus, not only did the practice of shielding-originally introduced as an emergency measure-remain unchallenged in many facilities for a very long time, but these developments also gave the impression that this practice had become part of a new normal in many places.^19^ As a comprehensive report among OECD countries shows,^20^ the process of transitioning from strict visiting bans to exceptions in “palliative situations” and enabling safe visits of essential care partners was accompanied by uncertainties and political inertia, but also institutional hesitancy to address *the* key ethical and legal issue of how fundamental rights for residents could be best secured.^21^ First and foremost, it was about the right to keep physically connected with core family members-of course, under the same safety precautions that apply to the staff daily entering the facility. This right is a genuine expression of the right to private and family life as stated by article 8 of European Convention on Human Rights.^22^ Instead, as the example of Switzerland shows,^6^ the societal and political environment tolerated to a large extent the practice of shielding, allowing residential care facilities to act on their own safety and liability considerations. In the light of legal and ethical frameworks regulating the application of fundamental rights including public health emergencies, the great silence on the protection of residents' fundamental rights or the unequal degree to which the needs of residents were publicly debated in many countries is surprising. Scholars have repeatedly identified this societal neglect, reinforced by the practice of shielding, as a form of ageism, which led to a structural stigmatization of residential care facilities during the pandemic.^24,25,26^ At the end of this development stood the conversion of the prevailing narrative of the *vulnerability of residents* in a society plagued by the pandemic to a *reverse vulnerability of society* by portraying residential care facilities as at-risk areas for the whole community.^26,27,28^ As an immediate consequence, also phenomena of stigmatization of healthcare workers could be observed.^29^ Importantly, both forms of vulnerability must be understood as expressions of the practice of shielding, with the second, “reverse” form being better able to explain the "great silence” mentioned above.

These findings increasingly put into perspective the dominant narrative of *personal* vulnerability, that is, of individuals who must be isolated to protect their lives. In contrast, they condense into a counternarrative of *spatial* vulnerability that concerns the places where care is provided as an independent and important structural determinant of viral transmission.^3,5^ Spatial vulnerability is not only added to, but exacerbates personal vulnerability, and consequently questions the validity of the justification provided for the practice of shielding: As the pandemic unfolded, the growing realization that the *place of care* intersectionally reinforces personal vulnerability confirmed many already known findings from social epidemiology.^8,10,30^ Beyond the neglect of social epidemiological knowledge, the long maintenance of the practice of shielding and the hesitant adaptation of visiting policies made it impossible to address the proportionality of ongoing restrictions on residents’ fundamental rights.^21,23^ Equally surprising was the scant attention paid to the structural determinants of intramural viral transmission: These include the availability of effective safety concepts, adequate protective equipment, a healthcare workforce in sufficient numbers, with sufficient skills and qualifications, able to respond effectively to the needs of residents.^31,32^

## Moral failure and the foreseeability of moral harm

In many places, the practice of shielding has been maintained for a long time although its defensibility in terms of effectiveness (with regard to intended and unintended outcomes) and proportionality (with regard to the means used to achieve desired ends) was increasingly doubtful.^4,6,7,8,18,20,30^ There is, of course, narrative evidence of numerous courageous managers who disregarded official visiting regulations and, within their range of possibilities, allowed safe and ongoing physical contact with residents already early in the pandemic and even in periods of high incidence in the general population. This included, for example, low-threshold on-site testing, same standards of personal protective equipment for visitors and healthcare workers, and tailored isolation policies limiting the extent of unavoidable isolation and quarantine measures to the minimum necessary. And, of course, there were many nurses and doctors who secretly granted exceptions to strict visiting rules, for example, by interpreting generously the term "palliative situation" (which justified an exception in many places), because they were emotionally overwhelmed by the suffering of residents or silently resisted the practice of shielding.^33^ As the example of Switzerland illustrates,^6,11^ this development was reinforced by the attitude of health authorities, which gave residential care facilities wide discretionary powers, often leaving managers no alternative but to stick to the practice of shielding for fear of liability issues.^6,12,13^ Most surprising, however, was the scant attention paid to the increasing evidence of the disastrous effects of prolonged visitation bans on residents’ lives and coupled with the very high Covid-19-related mortality of residents in many high-income countries.^7,8,20^ Although these phenomena demonstrated the increasing inadequacies of the widespread practice of shielding, they were not powerful enough to trigger a timely adaptation of this practice with the goal to minimize its known harmful effects. Along with the exacerbation of long-standing problems of “welfare conditionality”^34^ for older people in high-income countries^23^ and concomitant challenges for the residential care facility industry, these circumstances have been labeled a moral failure.^35,36,37^ Moral failure here can best be specified as a failure to timely adapt the justification of the practice of shielding at two levels:

• at a societal level by addressing manifestations of reverse vulnerability that might explain the “great silence” on guaranteeing the fundamental rights of residents and assessing the proportionality of their restriction under the conditions of a continuing pandemic.
• at an individual level by adopting the lens of social epidemiology on individual vulnerability and adequately considering the dimension of place as a crucial factor that exacerbates individual vulnerability and explains the doubtful effectiveness of visiting bans.^8^

These circumstances were characterized by upholding the practice of shielding in many places, even when its deleterious effects on the most vulnerable became increasingly apparent. Therefore, the failure to prevent foreseeable harm offers a first specification of moral failure.

## Moral failure and moral agency

In addition to considerations of preventing foreseeable harm, evaluating an agent’s action or omission as a *moral* failure requires further theoretical clarification. Above all, it presupposes that agents are considered *moral* agents, meaning that they are expected to give reasons for their choices and that these reasons should be based on ethical grounds: Judgments in the moral domain express moral obligations towards oneself or others, which ultimately serve to realize the *aretaically good* or the *epistemically right*. In motivational and cognitive terms, they presuppose both the willingness and the ability to be accountable for one’s own actions and omissions. Moral agency, then, can be pragmatically understood as a construct that bridges motivation, judgment, and reasoning with choice and subsequent action.^38,39,40^ However, in light of the possibility of moral failure, the talk of moral agency as being founded solely in the agent’s epistemic, moral, and practical qualities seems insufficient. The talk of moral failure (and, accordingly, also “moral success” as its opposite) presupposes not only that there is some instance or authority to which the agent is morally accountable, but also that-in accordance with the Kantian axiom that “ought implies can”^41^—the agent has a degree of freedom and choice as well as the physical capacities to act in the morally preferable way. Genuine choice should be based on well-reasoned ethical judgments that are intelligible, communicable, and verifiable for consistency.^41^ Thus, moral agency, in order to be fully realized, requires that agents have some degree of freedom in order to weigh options and enable an option to be chosen or rejected. In realizing moral agency, agents can encounter two types of limitations^36,42^:

1. First, the degree of freedom can be *too narrow*, preventing the agent from doing the (knowingly) morally preferable action, resulting in felt moral complicity.
2. Second, the degree of freedom can be *too broad* or indeterminate, requiring a choice between equally demanding ethical options, therefore entailing high risks both in terms of opportunity costs and unforeseeable harm, as a result of moral complexity.

The first limitation (i.e., a too low degree of freedom) is characterized by (internal or external) constraints that prevent the agent from doing the right action or force her to “cooperate in wrongdoing,”^43,44^ resulting in felt moral complicity.^42^ The latter limitation (i.e., a too broad degree of freedom), however, bears considerable moral risk, that is, the risk that the decision taken may turn out to be suboptimal or even wrong in retrospect, even though everything was done in advance to take all the known relevant aspects into account.^42^ Whereas felt moral complicity results in a psychological reaction for which Andrew Jameton coined the term moral distress,^43,45^ moral complexity can be considered a circumstance attributable to “veritable” moral dilemmas,^43^ that is, situations of “impossible” choice between options of equal worth, where each choice is (forced to be) associated with high opportunity costs because the benefits of (the) alternative choice(s) cannot be realized.

The importance of the degree of freedom that underlies a moral choice—whose antipodes are discussed with the concepts of moral distress and moral dilemma in the literature—completes the understanding of moral agency, and allows to shed a more critical light on the phenomenon of moral failure and to relate it to the practice of shielding in the context of residential care during the Coronavirus pandemic: In view of the societal mandate and significance of residential care for society, and of the obligation of these facilities to offer both shelter and care for members of society who need it, moral agency has to be understood both in the corporate and individual sense.^39^ It involves the ability of institutions and individuals to give reasons both for their actions and omissions, and to base these reasons on ethical grounds, that is, contributing in some way to the well-being of those who entrust themselves to their protection, thus realizing a good that is important for the entire community. For healthcare professionals, moral agency is anchored in codes of professional ethics, which form the basis for the profession’s trustworthiness and societal mandate. The attribution both of corporate and individual moral agency is important when it comes to the possibility of moral failure. Even if the further considerations relate to the individual dimension, the continued practice of shielding shows the close interconnectedness between individual and corporate moral agency.^39^ It adds the failure to acknowledge moral agency, that is, to act as moral agents, as a further specification of moral failure.

## Moral failure and moral character

In unfolding the justification of the practice of shielding, one of the premises consisted in the reasonability of public health measures (and concomitant restrictions on fundamental rights) in the presence of imminent danger, even without a sufficient evidence base. As for the aforementioned requirement of *reasonableness* in assessing the permissibility of restricting fundamental rights in situations of perceived public threat, proportionality requires that such measures be the least harmful, lawful in themselves, unavoidable to preserve a valuable public good, and justified by sufficient evidence.^46,47,48^ However, if evidence is not (yet) given, but the perceived threat to single populations or society as a whole in terms of serious and irreversible harm cannot be prevented with sufficient clearness, public health measures encompassing restrictions on fundamental rights might also be taken on a *precautionary* basis, that is, prior to the availability of evidence.^49^ Most importantly, it cannot be ruled out that these initial "cautious" measures may prove disproportionate in retrospect, that is, if evidence later emerges.^50^ Thus, from a character ethics perspective, the introduction of the practice of shielding at the *beginning* of the pandemic can be considered to be in line with the precautionary principle: It portrays health authorities and managers of residential care facilities as *prudent agents*, who—in light of the perceived threat of the Coronavirus both to residents and to the public—reacted to a *too broad* degree of freedom with strict, but prudent, measures consisting in a practice of shielding. In the light of the perceived threat and the initially listed psychological factors, it would be difficult to see how these *initial* measures could be characterized as moral failures, even if they may well be considered inappropriate when knowledge is added.^11^

Importantly, this differentiation no longer applies to the further developments that have not led to an adjustment of the practice of shielding, with novel knowledge being increasingly available, as well as better means of protection, and the evidence of the physical and psychological harm of the measures themselves: Insisting to following the virtue of prudence without addressing the issue of proportionality of the measures can be seen as a failure of character, as those in charge "held on" to the virtue of prudence against better judgment, by continuing to make "prudent decisions" that upheld the practice of shielding. As a consequence, in addition to understanding moral failure in terms of preventing foreseeable harm and (unacknowledged) moral agency, there is a third specification that focuses on requirements of moral character: In the context of visiting restrictions during the ongoing pandemic, moral failure is revealed as a *character failure* to substitute prudent choices for proportionate ones.

## Moral failure and moral practice

One of the consequences of reverse vulnerability—understood as a social phenomenon leading to the stigmatization of residential care facilities perceived as threats to society—has been the very late development of counterpractices to the practice of shielding to allow safe, continuous visits of essential care partners in the privately inhabited space of residents. This has been described as a threefold moral failure in terms of harm prevention, (unacknowledged) moral agency, and character failure in terms of persistence on prudential considerations. Due to the different stakeholders involved, this can be considered as a moral failure at different levels, that is, societal, political, institutional, but also personal. Health professionals were often the only ones who had ongoing access to residents and knew the impact of visiting bans on resident’s physical and mental health,^2,4,7^ but they did not necessarily consider themselves as moral agents entitled to address or question the practice of shielding, even though they experienced first line its deleterious effects.^29,33,51^

Regarding the ethical evaluation of this practice and its reference to moral character, Geoff Moore’s account of Alasdair MacIntyre’s virtue framework^52,53^ for the context of professional practice is clarifying: First, it basically contends that “practice” has to be considered a morally thick term insofar—in MacIntyre’s sense—it inherently produces morally valuable goods and agents have therefore to meet the requirements of moral character. Consequently, there cannot be such thing as a space of practice *outside* of moral agency. Second, it requires that not only personal, but also professional practices—in order to create morally valuable goods—meet requirements of character. Consequently, and building on MacIntyre’s *After Virtue*,^52^ Moore expands not only the concept of virtue to professional virtue, but also to virtuous organizations (Ref. [53], p. 112) which are distinguished by “organizational character.” Whereas the practice of virtue produces internal goods according to existing standards of excellence for the respective activity,^53^ (e.g., residential care), institutions are engaged with providing external goods that secure the framework conditions for internal goods to be achieved. However, “… unless the institution sustains pursuit of internal goods of the practice at its core, it is liable to decay from within” (Ref. [53], p. 112).

In this line of thinking, as the pandemic progressed, *upholding* the practice of shielding based on prudential considerations cannot meet the requirements for being considered as a practice in the moral sense, that is, a practice promoting the internal goods germane to residential care: It is precisely this understanding that offers a fourth specification of moral failure, that is, the failure to meet the standards of a moral practice. This occurred from the moment when external goods (such as the avoidance of lawsuits or lack of resources for visitor management) became more important than internal goods concerning the protection of the interests of residents. In contrast to the practice of shielding justified on the basis of prudential considerations, counterpractices ensuring proportionate measures would have better "fit" the available evidence,^54^ by considering both safety and quality-of-life considerations. For the four specifications of moral failure see Table 1.

**Table 1.** Four specifications of moral failure in the context of visiting restrictions in residential care after the first wave of the Covid-19 pandemic (source: author).

Dimension of reference	Specification of moral failure
1. Prevention of harm	Failure to prevent foreseeable harm
2. Moral agency	Failure to acknowledge moral agency
	a) At a personal level (professional code of ethics, professional autonomy, societal mandate of the profession)
	b) At an organizational level (mandate of the institution towards the welfare and interests of residents)
3. Moral character	Failure to adapt prudential considerations (at the beginning of the pandemic) with proportionality considerations (successive phases)
4. Moral practice	Failure to the align the external goods of residential care with the pursuit of internal goods

## Conclusion

As professional agents, healthcare professionals have been at the forefront not only of witnessing, but also applying the practice of shielding and implementing official or institutional regulations. Especially nurses in residential care witnessed the impact of social isolation on many residents, trying to compensate the loss of social connection, among others, by the use of social media.^29,33^ Against this background, it is difficult—if not presumptuous—to identify at what point, one can speak of a moral failure at an individual level. Additionally, the “heroization” of nurses and other healthcare professionals in the pandemic can be assumed to have complicated the focus on issues of moral agency in the light of exacerbated resident vulnerability and intergenerational social justice.^51,55^ In the meanwhile, there is growing evidence of the ethical burden carried by healthcare professionals including moral distress (e.g.,^29,56^). Following the narrow definition, moral distress can be understood as a psychological reaction following the experience of moral complicity.^45,42^ In this line of thinking, the phenomenon of moral distress lends itself well to showing the psychological impact in individuals of what has so far been described as moral failure in all its specifications (see Table 1).

Departing from the impact of the Coronavirus pandemic on residential care in high-income countries, immediate responses to the emerging vulnerability of residents have been described as constituents of a practice of shielding. In the successive pandemic waves, this practice, motivated by prudential considerations, has proved more and more to be both ineffective and bear considerable harm, but also to lack considerations of proportionality. These developments, together with phenomena of societal stigmatization of residential care, expressed by the term of “reverse vulnerability,” have been described as manifestations of ageist thinking. Not only have they progressively invalidated the justification of the practice of shielding, but they have also been interpreted as elements that explain moral failure. On a normative level, the appropriateness of speaking of moral failure has been unfolded in the four dimensions of harm prevention, moral agency, moral character, and moral practice in MacIntyre’s sense^52^ (see Table 1). At the level of healthcare professionals, the possibility of moral failure has been related to the high prevalence of moral distress in healthcare professionals during the pandemic,^56^ which can be considered as a descriptor of the psychological impact of moral failure on individuals.

Although the pandemic threat of the Coronavirus appears to have been contained at the time of writing, the risks of new emerging threats and related public health crises cannot be readily averted. They are likely to impact known or novel populations at risk and to exacerbate once again gradients of vulnerability. With respect to residential care, the often raised call for “ethical preparedness”^57^ during the pandemic implies that professionals and facilities demonstrate moral character, both through prudent and proportionate choices. But “ethical preparedness” is also shown through moral courage,^55^ understood here as the ability to resist pressures from external goods that have proven to be serious obstacles to the achievement of internal goods, thus threatening the inherent moral significance of practice within residential care. Such endeavor can be considered as a manifestation of both personal and institutional moral resilience.^58^

In strengthening moral resilience, healthcare professions’ education plays a pivotal role by early fostering in students the ability and willingness to respond to ethical challenges in public health crises. Promising approaches to ethics teaching involve overcoming the “limitation” of moral virtue to the dimension of the private, but also avoiding strict separations between moral and civic virtues in the context of ethics teaching.^59,60^ However, more conceptual and empirical research is needed to explore the potential of ethics education to reduce experiences of moral failure and improve ways to deal with it effectively, in order to facilitate healthcare students’ early identification as trusted members of a caring profession within a caring society.
